# Differential suppression of persistent insect specific viruses in trans-infected *w*Mel and *w*MelPop-CLA *Aedes*-derived mosquito lines

**DOI:** 10.1016/j.virol.2018.11.012

**Published:** 2019-01-15

**Authors:** Breeanna J. McLean, Kimberley R. Dainty, Heather A. Flores, Scott L. O’Neill

**Affiliations:** Institute of Vector-Borne Disease, Monash University, Clayton, Vic., Australia

**Keywords:** *Wolbachia*, Flavivirus, Bunyavirus, Insect specific viruses, Mosquito cell lines

## Abstract

*Wolbachia* suppresses the replication of +ssRNA viruses such as dengue and Zika viruses in *Aedes aegypti* mosquitoes. However, the range of viruses affected by this endosymbiont is yet to be explored. Recently, novel insect-specific viruses (ISVs) have been described from numerous mosquito species and mosquito-derived cell lines. Cell-fusing agent virus (*Flaviviridae*) and Phasi Charoen-like virus (*Bunyaviridae*) persistently infect the *Ae. aegypti* cell line Aag2 which has been used for experimental studies with both the *w*Mel and *w*MelPop-CLA strains. *Wolbachia* was found to restrict the replication of CFAV but not the PCLV infection in these lines. Furthermore, an additional *Ae. albopictus* cell line (RML-12) which contained either *w*Mel or *w*MelPop-CLA was assessed. While no infectious +ssRNA or dsRNA viruses were detected, a PCLV infection was identified. These observations provide additional evidence to support that insect-specific, +ssRNA viruses can be suppressed in cell culture by *Wolbachia* but -ssRNA viruses may not.

## Highlights

• *Wolbachia* suppresses CFAV in Aag2 *w*Mel and *w*MelPop-CLA cell lines.
• PCLV persistently infects the RML-12 cell line.
• *lbWo*LA c*achia* does not suppress PCLV in Aag2 or RML-12 *w*Mel and *w*MelPop-Cell lines.

## 1 Introduction

Arthropod-borne viruses are a diverse group of mosquito, sand-fly and tick transmitted pathogens responsible for significant diseases in humans. While traditional control methodologies have been based primarily on vector population suppression and vaccine development, focus has recently been placed on finding long term, cost-efficient and sustainable options for disease control. The intracellular endosymbiont *Wolbachia* has been shown to significantly suppress the replication of a broad range of viruses in *Drosophila* models and interestingly retained this pathogen blocking phenotype upon trans-infection into both *Aedes*-derived cell lines and *Aedes aegypti* mosquitoes (Aliota et al., 2016, Moreira et al., 2009, Teixeira et al., 2008, Walker et al., 2011). Fields trials in which *Wolbachia-*infected mosquitoes have been released to facilitate *Wolbachia* invasion of resident *Ae. aegypti* populations are currently underway in a number of countries (Dutra et al., 2015, Hoffmann et al., 2011, Nguyen et al., 2015) with the aim of utilizing the virus blocking properties of *Wolbachia* to reduce arbovirus transmission to people. However, while a significant amount of data has been accumulated on the effects of *Wolbachia* on transmission of major arboviruses from the *Flaviviridae* and *Togaviridae* families, relatively little is known about the taxonomic limits of virus blocking by *Wolbachia* (Aliota et al., 2016, Glaser and Meola, 2010, Moreira et al., 2009, Shaw et al., 2012). To date, few studies have investigated the effect of co-replication of *Wolbachia* and negative sense single stranded RNA (–ssRNA) (Glaser and Meola, 2010, Parry and Asgari, 2018, Schultz et al., 2018) or double stranded RNA (dsRNA) viruses (Shaw et al., 2012) in mosquito hosts. Furthermore, as the mechanism of *Wolbachia*-mediated suppression remains poorly understood, +ssRNA viruses which display altered tropism and transmission strategies to that of other pathogenic viruses of the same family are of interest to be assessed for suppression by *Wolbachia*.

With the advent of novel and more effective sequencing technologies, an increasing number of divergent, maternally transmitted mosquito-borne viruses are being described (Blitvich and Firth, 2015, Hall et al., 2016). These insect-specific viruses (ISVs) are hypothesised to form life-long, persistent viral infections within their host primarily transmitted vertically between generations (Blitvich and Firth, 2015). While the intricacies of tissue tropism are yet to be fully investigated, preliminary findings have suggested localisation of ISVs in reproductive tissues with infection of progeny either occurring during embryo development or oviposition (Bolling et al., 2011, Hall-Mendelin et al., 2016). Similarly, analysis of *Wolbachia*-specific tropisms in *Ae. aegypti* mosquitoes have shown high density infections in reproductive tissues (Frentiu et al., 2014, Walker et al., 2011) suggesting that in a co-infected mosquito, ISVs and *Wolbachia* have the potential to interact. While there has been limited studies in mosquitoes to investigate this interaction (Amuzu et al., 2018), the *Ae. aegypti* derived cell line Aag2 which has been routinely utilised in *Wolbachia*-related research (Hussain et al., 2013, Lu et al., 2012) has been shown to be persistently infected with two ISVs; Cell fusing agent virus (CFAV; *Flaviviridae*) and Phasi Charoen-like virus (PCLV; *Bunyaviridae*) (Cammisa-Parks et al., 1992, Maringer et al., 2017, Stollar and Thomas, 1975). Interestingly, the first report of CFAV, a +ssRNA virus, originated from this cell line as a viral agent responsible for severe cytopathic effects when supernatant harvested from these cells was inoculated onto an *Ae. albopictus* line (Stollar and Thomas, 1975). Further characterisation identified this agent as a divergent insect-specific flavivirus that has been subsequently isolated from both *Ae. aegypti* wild caught and laboratory colony material (Bolling et al., 2015, Cammisa-Parks et al., 1992, Cook et al., 2006). PCLV, a –ssRNA virus of the newly proposed insect-specific *Goukovirus* genus was initially isolated from *Ae. aegypti* mosquitoes from Thailand. However, recently, it was also shown to persist in the Aag2 cell line alongside CFAV (Chandler et al., 2014, Maringer et al., 2017, Marklewitz et al., 2011).

To determine if the introduction of *Wolbachia* to Aag2 cells impacted the replication of these viruses, analysis undertaken by Schnettler et al. (2016) and Zhang et al. (2016) examined Aag2 cells stably-infected with *w*MelPop-CLA for the presence of CFAV and PCLV by RT-PCR. It was suggested by both studies that *Wolbachia* severely restricts the replication of CFAV. However, upon tetracycline treatment of these lines to clear *Wolbachia,* contradictory results were obtained where one study suggested CFAV replication could recover after the removal of *Wolbachia* while the other confirmed clearance of the infection. Interestingly, it was demonstrated that *Wolbachia* had no effect on the insect-specific bunyavirus (PCLV) known to persistently infect these lines (Schnettler et al., 2016). To address the extent of *Wolbachia*-mediated suppression on ISVs and more broadly the effect of this bacterium on mosquito cultures persistently infected with +ssRNA and –ssRNA viruses, mosquito cell cultures (Aag2 and RML-12) stably infected with *w*Mel and *w*MelPop-CLA were assessed for CFAV and PCLV replication both prior to and following tetracycline treatments. CFAV replication was not detected in *Wolbachia* infected cultures nor was the virus able to be recovered upon passage in the highly permissive RNAi-deficient *Ae. albopictus* (C6/36) cell line. Conversely, PLCV was detected and isolated from both *Wolbachia* infected cell lines.

## 2 Results

### 2.1 *w*Mel and *w*MelPop-CLA suppresses CFAV in Aag2 cell line

**Fig. 1:**
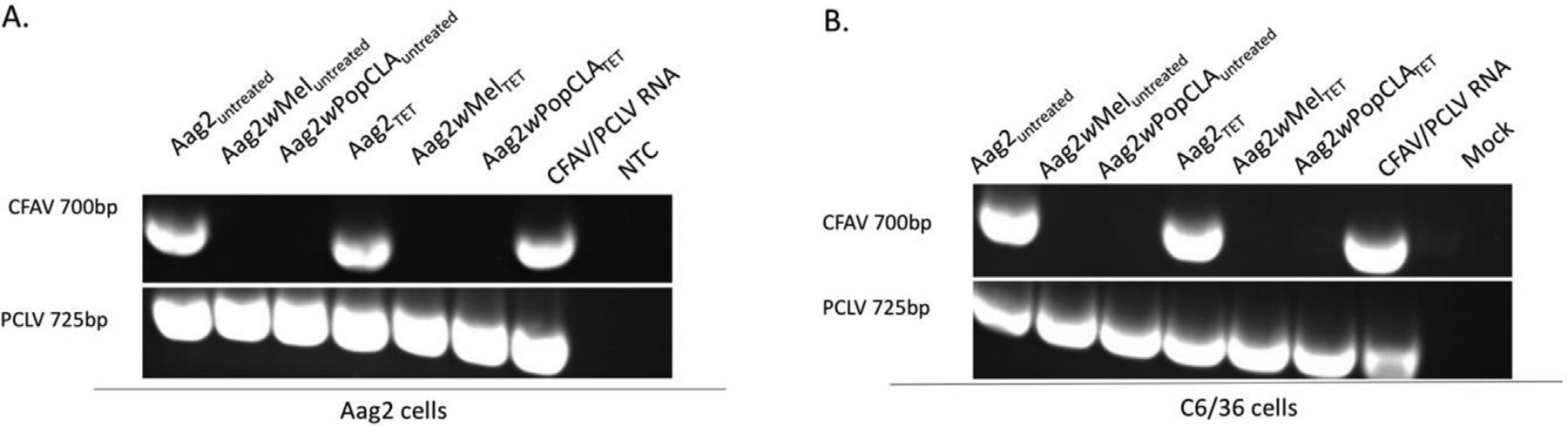
Effect of *Wolbachia* on CFAV and PCLV in Aag2 cell lines and passaged C6/36 supernatant. CFAV or PCLV detected in supernatant by RT-PCR in (A) *Wolbachia* untreated or tetracycline treated Aag2 lines and (B) *Wolbachia* untreated or tetracycline treated passage 1 supernatant collected from C6/36 cells. CFAV/PCLV RNA was derived from a virus isolate passaged twice on C6/36 cells confirmed to contain both CFAV and PCLV. ‘NTC’ represents a no template control while ‘Mock’ represents RNA extracted from passaged maintenance media on C6/36 cells.

The *Ae. aegypti* cell line Aag2 has been shown to be infected with two insect-specific viruses (ISVs) (Maringer et al., 2017, Scott et al., 2010). To determine the effect of co-replicating *Wolbachia* on the insect-specific flavivirus CFAV, supernatant was harvested from *Wolbachia*-free Aag2 cells as well as Aag2 cells infected with *w*Mel or *w*MelPop-CLA and screened by RT-PCR (Fig. 1A). While CFAV replication was detected in the control Aag2 *Wolbachia*-free line, no replication was detected in cells persistently infected with *Wolbachia* at either time point. To further elucidate if this was due to *Wolbachia* actively supressing this virus, cell lines were treated with tetracycline for 3 successive passages. The presence of infectious CFAV was not detected by ELISA in tetracycline-treated *w*Mel or *w*MelPop-CLA lines upon inoculation or an additional passage of supernatant on C6/36 cells (Table 1). Absence of flavivirus replication was further confirmed by CFAV-specific RT-PCR from supernatant harvested from passage 1 (Fig. 1B).

**Table 1:** Detection of CFAV by anti-dsRNA ELISA in Aag2 cells.

	Aag2_UNTREATED_		Aag2_TET_		Aag2 *w*Mel_UNTREATED_		Aag2 *w*Mel_TET_		Aag2 *w*MelPop-CLA_UNTREATED_		Aag2 *w*MelPop-CLA_TET_	
	CPE	3G1 ELISA#	CPE	3G1 ELISA#	CPE	3G1 ELISA#	CPE	3G1 ELISA#	CPE	3G1 ELISA#	CPE	3G1 ELISA#
P0	+	+	+	+	–	–	–	–	–	–	–	–
P1	+	+	+	+	–	–	–	–	–	–	–	–

Legend + positive; - negative.

Cytopathic effect (CPE) was observed on these samples during analysis.

# Fixed-cell ELISA using the anti-dsRNA mAb 3G1 against 4, C6/36 wells inoculated with supernatant from Aag2 with or without *Wolbachia*. Samples deemed positive by OD> 0.11.

### 2.2 Wolbachia does not affect PCLV replication in co-infected Aag2 cells

To determine if *Wolbachia* supressed the insect-specific bunyavirus PCLV in co-infected Aag2 cell culture, supernatant obtained from *Wolbachia*-free Aag2 cell and Aag2 cells infected with *w*Mel or *w*MelPop-CLA was screened by RT-PCR. In contrast to what was observed with CFAV, PCLV was detected in the *Wolbachia*-free Aag2 line as well as both *Wolbachia* infected lines. To further confirm if this was an active viral infection capable of infecting new cells, supernatant harvested from both *Wolbachia* untreated and tetracycline-treated lines was inoculated onto the permissive C6/36 line for 2 successive passages. PCLV replication was confirmed by RT-PCR in C6/36 supernatant obtained from passage 1 suggesting infectious PCLV was produced in the Aag2 line.

### 2.3 PCLV replication detected in *w*Mel and *w*MelPop-CLA infected RML-12 cell lines

**Fig. 2:**
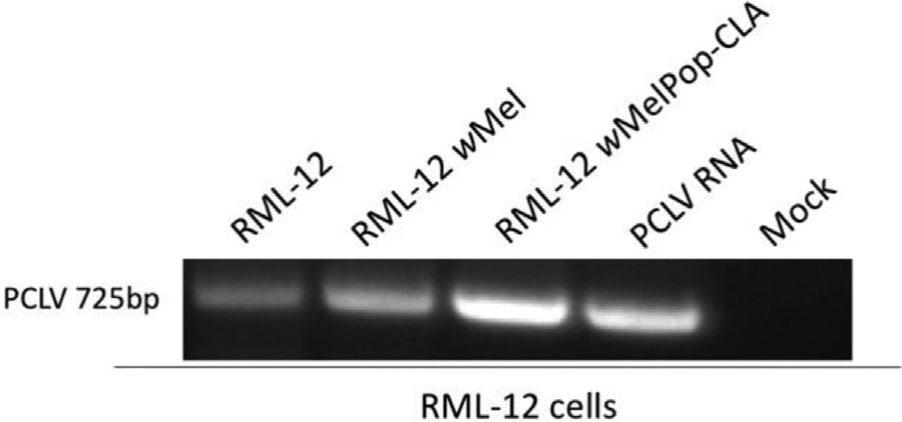
RT-PCR detection of PCLV in supernatant harvested from *w*Mel, *w*Pop-CLA or *Wolbachia*-free RML-12 cell lines. PCLV RNA used for analysis derived from a confirmed PCLV isolate passaged twice on C6/36 cells. ‘Mock’ represents RNA extracted from passaged maintenance media on C6/36 cells.

To investigate the potential of PCLV to be detected in other *Wolbachia* infected cell lines, *Ae. albopictus*, *Wolbachia*-free RML-12 cells as well as RML-12 cells stably infected with *w*Mel and *w*MelPop-CLA were assessed. PCLV was detected by RT-PCR in supernatant from RML-12 lines regardless of W*olbachia* infection (Fig. 2). All three cell lines were assessed for CFAV infection by RT-PCR and found to be uninfected (Fig. 2). Furthermore, to determine if either +ssRNA or dsRNA viruses were present, C6/36 monolayers infected with supernatant harvested from these lines was assessed by fixed cell ELISA. No reactivity was observed in passage 0 or passage 1 using the anti-dsRNA mAb 3G1 demonstrating that this cell line was not persistently infected by these virus types (Table 2).

**Table 2:** Detection of virus by anti-dsRNA ELISA in RML-12 cells.

	RML-12 wt		RML-12 *w*Mel		RML-12 *w*MelPop-CLA	
	CPE	3G1 ELISA#	CPE	3G1 ELISA#	CPE	3G1 ELISA#
P0	–	–	–	–	–	–
P1	–	–	–	–	–	–

Legend + positive; - negative.

Cytopathic effect (CPE) was observed on these samples during analysis.

# Fixed-cell ELISA using the anti-dsRNA mAb 3G1 against 4, C6/36 wells inoculated with supernatant from RML-12 cells with or without *Wolbachia*. Samples deemed positive by OD> 0.5145.

## 3 Discussion

The endosymbiont *Wolbachia* been widely studied for its pathogen blocking phenotype against +ssRNA viruses (Aliota et al., 2016, Chrostek et al., 2013, Ferguson et al., 2015, Glaser and Meola, 2010, Moreira et al., 2009, Osborne et al., 2012). However, co-infection of *Wolbachia* with viruses which exhibit different transmission (vertical or venereal) and replication strategies (–ssRNA or dsRNA) in mosquitoes are yet to be widely explored. Recently, ISVs have been isolated in high prevalence from both wild type and laboratory-reared mosquitoes (Blitvich and Firth, 2015, Bolling et al., 2015, Hall et al., 2016). These isolations form an interesting addition to arboviral research as *in vitro* and *in vivo* investigations into the impact of these viruses within the mosquito virome have demonstrated that ISVs can either enhance or suppress the replication of closely related, medically significant pathogens (Bolling et al., 2012, Hall-Mendelin et al., 2016, Newman et al., 2011). The mosquito species *Ae. aegypti* is not only a significant vector of human pathogens DENV, Zika virus and Yellow fever but also a known carrier of the ISVs CFAV and PCLV (Cook et al., 2006, Yamao et al., 2009). As *Ae. aegypti* is the focus of *Wolbachia* related research in mosquitoes (Ferguson et al., 2015, Frentiu et al., 2014, Moreira et al., 2009) it is likely that this bacterium is not only interacting with viruses known to cause disease but also with the ISVs that persistently infect this species.

To further assess these interactions between *Wolbachia* and viruses which persistently infect mosquitoes, Aag2 *w*Mel and *w*MelPop-CLA cell lines were evaluated for the presence of two ISVs (CFAV and PCLV) previously shown to replicate within the parental Aag2 cell line (Maringer et al., 2017, Stollar and Thomas, 1975). The replication of CFAV, a member of the *Flaviviridae* family, was restricted by *Wolbachia* as this virus could not be detected by RT-PCR in supernatant harvested from Aag2 *w*Mel and *w*MelPop-CLA lines. These results are consistent with studies based on RT-PCR alone by Schnettler et al. (2016) which reported suppression of CFAV in the *w*MelPop-CLA line. As the presence of infectious CFAV had not been described by previous studies, it was assessed by the inoculation of permissive, *Ae. albopictus* (C6/36) cells with supernatant harvested from the Aag2 line with or without *Wolbachia*. CFAV was not detected by anti-dsRNA ELISA from passaged, *w*Mel, *w*MelPop-CLA or matched tetracycline treated lines. This study and Schnettler et al. (2016) both contrast the observations reported in Zhang et al. (2016) as infectious CFAV could not be recovered after tetracycline treatment. As density is a well described factor contributing to *Wolbachia*-mediated suppression, Zhang et al. (2016) hypothesised that the difference in CFAV replication may have been due to lower *Wolbachia* densities maintained in the line. However, as it's likely that the CFAV infection was lost prior to both the screening by this study and that of Schnettler et al. (2016), it is difficult to determine how density affects the replication of persistent ISF infections in cell lines without observations over multiple passages.

Viral clearance of the insect-specific, -ssRNA bunyavirus PCLV was not observed in either *w*Mel- or *w*MelPop-CLA-infected Aag2 or RML-12 lines. Consistent with Schnettler et al. (2016), PCLV was detected by RT-PCR in supernatant harvested from *Wolbachia*-infected Aag2 cells and in inoculated C6/36 cells. (Fig. 1A and B). Furthermore, additional assessment of *Aedes* derived RML-12 cell lines containing *Wolbachia*, found that all cell lines were persistently infected with PCLV but no dsRNA or +ssRNA viruses. As PCLV has only been identified from *Ae. aegypti* mosquitoes and the RML-12 line was reported to have originated from this species it is possible that like CFAV, the viral infection was established at the generation of the line (Chandler et al., 2014, Maringer et al., 2015, Peleg, 1968, Stollar and Thomas, 1975). However, recent analysis by McLean et al. (2015) and Voronin et al. (2010) which demonstrated that the RML-12 line was derived from *Ae albopictus* may contradict this hypothesis as it suggests that the line may have been either initially misidentified or was contaminated with a second cell type during passage. While the permissibility of the RML-12 line to persistent PCLV infection raises further investigation about *Ae. albopictus* as a natural reservoir for this virus, the inability of *w*Mel and *w*MelPop-CLA strains to efficiently clear co-infected cultures suggest that PCLV evades *Wolbachia* by mechanisms associated with the replication strategies of this virus rather than by species-specific interactions.

Interestingly, this and Schnettler et al. (2016) are not the only reports of *Wolbachia* failing to supress replication of -ssRNA viruses. Schultz et al. (2018) determined that the replication of La Crosse virus (LACV; *Bunyaviridae*) and vesicular stomatitis virus (VSV; *Rhabdoviridae*) was not affected by *Wolbachia* (*w*Stri) in *Ae. albopictus* cells while Parry and Asgari (2018) demonstrated that endogenous *Aedes* anphevirus (AeAV; order *Mononegavirales*) could be isolated from both *w*MelPop-CLA and *w*MelPop-CLA_TET_ cell lines. Furthermore, *in vivo* studies by Glaser and Meola (2010) demonstrated that *Wolbachia* did not suppress the replication of LACV in co-infected *Drosophila melanogaster* and more recently Dodson et al. (2017) reported that the transmission of Rift Valley fever virus (RFV; *Bunyaviridae*) was not restricted by *Wolbachia* in *Culex tarsalis*. Taken in combination, this provides mounting evidence to suggest differential viral interactions with *Wolbachia* whereby +ssRNA viruses are susceptible to suppression but –ssRNA viruses may not. However, while *Wolbachia* restricted CFAV replication, additional investigation should be undertaken in *Ae. aegypti* mosquitoes to confirm that –ssRNA viruses and +ssRNA viruses which display different tropism and transmission strategies are suppressed by *Wolbachia in vivo*. The mechanisms underlying viral suppression remain unresolved. Investigations into the interactions of *Wolbachia* with other virus types may provide information critical to understand the impact of *Wolbachia* on the mosquito virome as well as mechanisms of viral blocking.

## 4 Conclusion

In summary, *Wolbachia* restricts the replication of the +ssRNA virus, CFAV, but not –ssRNA virus, PCLV, in co-infected cell culture. These results support previous studies which have suggested similar interactions (Schnettler et al., 2016, Zhang et al., 2016) while also confirming the viral clearance of CFAV by *Wolbachia* as no infectious virus was detected by anti-dsRNA ELISA. As ISVs display tropisms and transmission strategies generally not associated with other pathogenic arboviruses, further investigation is required to determine if this effect is observed in *Ae. aegypti* mosquitoes. Interestingly, to date, *Wolbachia* has not been shown to affect the replication of –ssRNA viruses (Dodson et al., 2017, Glaser and Meola, 2010, Parry and Asgari, 2018, Schultz et al., 2018) suggesting a new avenue to explore *Wolbachia*-mediated interference which may extend our understanding of the underlying mechanism of interference.

## 5 Methods

### 5.1 Cell line maintenance

C6/36 and RML-12 (*Ae. albopictus*) cells were cultured at 28 °C in RPMI 1640 medium (C6/36) or Schneider's Drosophila/Mitsuhashi and Maramorosch 50:50 medium (RML-12) supplemented with 10% foetal bovine serum (FBS) and L-glutamine. *Ae. aegypti* Aag2, Aag2 *w*Mel and Aag2 *w*MelPop-CLA cells were cultured in Schneider's/Grace's insect medium (50:50) containing 10% FBS and L-glutamine at 26 °C. Tetracycline treated lines were generated from Aag2, Aag2 *w*Mel and Aag2 *w*MelPop-CLA by the addition of 10 μg/ml of tetracycline to the maintenance media for 3 successive passages.

### 5.2 Virus identification and isolation from mosquito cell lines

Following the final tetracycline (tet)-treatment of Aag2, Aag2 *w*Mel and Aag2 *w*MelPop-CLA lines, supernatant from each untreated and tet-treated line was harvested. Two hundred microliters of supernatant from each *Wolbachia* untreated and tet-treated line as well as medium used to culture Aag2 (mock) was inoculated onto semi-confluent C6/36 cells and incubated at 28 °C for 5–7 days. Supernatant was harvested and immediately passaged onto fresh C6/36 cells for an additional passage, with the remaining supernatant used for RNA extraction. Monolayers were then fixed using 4% formaldehyde/0.5% Triton X for 4 h at 4 °C. To determine if ISVs were present in other *Aedes*-derived, *Wolbachia*-infected lines, RML-12 cells (wt, *w*Mel or *w*MelPop-CLA) were assessed (with the exception of tetracycline treatment) by the methods described above. Fixed-cell ELISA was performed using the anti-dsRNA mAb 3G1 as per previously described methods (O'Brien et al., 2015). RNA was extracted from 100 μl of culture supernatant of all samples using the RNeasy mini kit (Qiagen). Five-microliters of purified RNA was then screened by RT-PCR (SuperScript III One-Step RT-PCR System with Platinum Taq DNA) using CFAV and PCLV-specific primers from Schnettler et al. (2016). As additional confirmation of virus isolation, RNA was also extracted directly from supernatant harvested from Aag2, Aag2 *w*Mel and Aag2 *w*MelPop-CLA cell lines.
